# MiR-190a potentially ameliorates postoperative cognitive dysfunction by regulating *Tiam1*

**DOI:** 10.1186/s12864-019-6035-0

**Published:** 2019-08-22

**Authors:** Qiang Liu, Aisheng Hou, Yongyi Zhang, Ying Guo, Jingjing Li, Yinghao Yao, Kaimeng Niu, Hao Li, Yunlong Ma, Jiangbei Cao

**Affiliations:** 1 State Key Laboratory for Diagnosis and Treatment of Infectious Diseases, The First Affiliated Hospital, Collaborative Innovation Center for Diagnosis and Treatment of Infectious Diseases, Zhejiang University School of Medicine, Hangzhou, China; 2 Anesthesia and Operation Center, Chinese PLA General Hospital, Beijing, China; 3 Department of Anesthesia, Chinese PLA No.211 Hospital, Harbin, China

**Keywords:** POCD, miR-190a, Neurocognitive disorders, Brain, Animal model

## Abstract

**Background:**

Postoperative cognitive dysfunction (POCD) affects a large number of post-surgery patients, especially for the elderly. However, the etiology of this neurocognitive disorder is largely unknown. Even if several studies have reported a small number of miRNAs as the essential modulatory factors in POCD, these findings are still rather limited. The aim of current study was to screen the POCD-related miRNAs in the hippocampus tissues and investigate the target genes of differentially expressed miRNAs and their biological functions underlying POCD pathophysiology.

**Methods:**

The miRNA microarray was used to find the abnormal expression of miRNAs in the hippocampus tissues from the POCD model mice to normal mice (Discovery cohort, 3 vs 3). The nominal significant results were validated in an independent sample of hippocampus tissues of 10 mice based on same miRNA microarray (Replication cohort, 5 vs 5). Expression level of the most significantly abnormal miRNA was further validated by real-time quantitative polymerase chain reaction (PCR). To determine the expression pattern among miRNAs and genes and detect the interactions, we conducted a weighted gene co-expression network analysis (WGCNA) in the miRNAs and genes expression data from hippocampus tissue of wild type mice (*n* = 24). The target genes of miRNAs were predicted using the miRWalk3.0 software. Furthermore, we used the ClueGO software to decipher the pathways network and reveal the biological functions of target genes of miRNAs.

**Results:**

We found that nine miRNAs showed significant associations with POCD in both datasets. Among these miRNAs, mmu-miR-190a-3p was the most significant one. By performing WGCNA analysis, we found 25 co-expression modules, of which mmu-miR-190a-3p was significantly anti-correlated with red module. Moreover, in the red module, 314 genes were significantly enriched in four pathways such as axon guidance and calcium signaling pathway, which are well-documented to be associated with psychiatric disorders and brain development. Also, 169 of the 314 genes were highly correlated with mmu-miR-190a-3p, and four genes (*Sphkap*, *Arhgef25*, *Tiam1*, and *Ntrk3*) had putative binding sites at 3′-UTR of mmu-miR-190a-3p. Based on protein-protein network analysis, we detected that *Tiam1* was a central gene regulated by the mmu-miR-190a-3p.

**Conclusions:**

Taken together, we conclude that mmu-miR-190a-3p is involved in the etiology of POCD and may serve as a novel predictive indicator for POCD.

**Electronic supplementary material:**

The online version of this article (10.1186/s12864-019-6035-0) contains supplementary material, which is available to authorized users.

## Background

Postoperative cognitive dysfunction (POCD), a commonly-seen postoperative complication especially for elderly patients (age > 65-year-old), is characterized by cognitive impairments in patients underwent major surgery, and associated with high morbidity and mortality [1]. It covers a wide range of cognitive dysfunctions including short or long-term memory loss and attention deficit [2]. POCD was reported to occur in 25.8% of the patients seven days post-surgery, and in 12.7% of the patients 3 months post surgery [3–5]. This indicates that when the early postoperative neurocognitive disorders happen, the human body simultaneously initiates the self-repair mechanism for improving or restoring the cognitive function [6]. However, the mechanisms remain to be fully elucidated. Since POCD often leads to prolonged hospital stays, decreased quality of life, and increased social dependence as well as medical expenses [3], it is important to elucidate its underlying molecular mechanisms for the prevention, diagnosis, and treatment of POCD.

MicroRNAs (miRNAs), which are extensively distributed in eukaryotes, regulate expression of genes by 1) combining with 3-UTR of target gene to inhibit the translation or to degrade target mRNAs at the post-transcription level; and 2) interacting with other target non-coding RNAs, e.g. long non-coding RNA (lncRNA) or circular RNA (circRNA) [7, 8]. A great number of miRNAs enriched in human brain has been shown to express in a developmental stage-specific, tissue-specific, and cell-specific pattern [9] and play crucial roles in development of the neural system and in cognitive process such as learning and memorizing [10, 11]. Recently, multiple lines of evidence have demonstrated that miRNAs are involved in the pathogenesis of several neuropsychiatric diseases [11–15]. For example, based on genome-wide transcription sequencing, Liu et al. demonstrated that miRNAs and lncRNAs were important contributors in the pathogenesis of schizophrenia [14]. Furthermore, multiple aberrantly expressed miRNAs were implicated in the development of Huntington’s disease [13], neural tube defects [16], and Alzheimer’s disease [17]. As for POCD, several miRNAs have been reported to be associated with it [6, 18–20], such as, Yu et al. [6] reported that miR-572 was implicated in the development and restoration of POCD and might serve as a biological marker in early diagnosis of POCD. However, the specific roles and underlying mechanism of miRNAs in POCD are still not yet clear.

Hence, in this study, by focusing on the biology of the dysregulated miRNAs in POCD, we conducted a comprehensive analysis of miRNAs expression profiles in hippocampus tissue of POCD model mice, and made further investigation of the miRNAs’ potential interaction mechanisms with target genes. Our findings not only extend the understanding of miRNAs’ role in the pathogenesis of POCD but also facilitate the identification of the miRNA biomarkers for improving the prognosis of POCD patients.

## Methods

### Animals

Healthy aged male C57BL/6 mice (*n* = 22, aged 18 months, weights from 40.2 g to 46.6 g) were purchased from SiBeiFu Experimental Animal Science and Technology Co. Ltd. (Beijing, China. Permit Number: SCXK (Jing) 2016–0002). The mice were individually housed in an air-conditioned room with a temperature of 24 ± 4 °C and 55–65% humidity, under a standard 12 h–12 h light-dark cycle (lights on 6 AM to 6 PM), and had free access to standard food and water. The mice were acclimatized for 1 week before the experiment. The protocol of animal experiment was approved by the Animal Care Committee of the Chinese People’s Liberation Army General Hospital (Beijing, China).

According to the protocol of our research work, all mice were euthanized before we were prepared to obtain hippocampal tissue. When those mice scheduled for obtaining the hippocampal tissue, one by one, they were put into an anesthetic introduction chamber and were anesthetized with isoflurane into a deeper level. The introduction chamber was kept clean to minimize the odor that might distress animals subsequently anesthetized. A rodent anesthesia machine were used (Model: ABM09–002, Reward, Shenzhen) and the anesthetic used was isoflurane. The concentration of the vaporizer was set at 3% and the oxygen flow rate was set at 3 L/min during anesthesia. All mice were deeply anesthetized based on following signs: the slowed rising and falling of chest, no respond to toe pinch, and corneal reflex disappeared. Then, the mice were decapitated by using a guillotine in a uniformly instantaneous manner. The brain was instantly dissected on ice, and the hippocampal tissue was obtained and put into liquid nitrogen. All the mice were decapitated 24 h after operation. The detailed time line of the euthanization of study animals is shown in the Additional file 2: Figure S1.

### Tissue sampling

Mice were numbered by weight and randomly divided into two groups: Surgery and Sham, with 11 mice per group. The mice in the Surgery group were exposed to abdominal surgery under local bupivacaine anesthesia (according to the protocol from Xu et al. [21]), whereas those in the Sham group did not suffer from the anesthesia and surgery. At 24 h after the surgery, three mice were randomly selected from each group and sacrificed. The hippocampus tissue was removed immediately and stored in the sterile tube (RNase Free) in liquid nitrogen.

### Morris water maze (MWM)

The MWM test, as a hippocampal-dependent test, was applied to evaluate the spatial learning, spatial memory and cognitive flexibility for the mice [22]. The cognitive function of remaining 8 mice in each group were assessed by the MWM experiment. The water maze was a black circular tank (120 cm in diameter and 50 cm in depth) and filled with water of 22 ± 1 °C to a depth of 35 cm. Several visual objects were installed above the pool to help mice identify the direction. The maze was divided into four quadrants, an invisible platform (10 cm in diameter) was placed 1.5 cm blew the water surface in the first quadrant (target quadrant). The whole experiment was performed under a dark and quite environment.

During the experiment, mice were released into the water facing the wall of the tank from one of the four quadrants. The mice were trained to find the hidden platform and climb onto it within 60 s. The animals were allowed to stay on the platform for at least 10 s after each trial. If the mice were unable to find the platform in 60 s, it was then placed on the platform for 10 s. After that, the mice were put back to the cage and the second mouse was tested on trial 1. This rotation was repeated until all animals completed trial 1. Subsequently, the process was repeated for subsequent trials until 4 trials completed per day for 5 consecutive days. At 6th day, the platform was removed, and the mice were sent to evaluate their reference memory by being released from the third quadrant. The swimming speed, platform-site crossing numbers, dwelling time in the target quadrant and the escape latency were recorded.

### RNA extraction

Total miRNAs were extracted from the hippocampal tissue (*n* = 3 from each group) by the miRcutemiRNA kit (TIANGEN, DP501). The nanodrop was utilized to determine the miRNA concentration of each sample according to the optical absorption at 260 nm and the gel electrophoresis was used to detect the miRNA integrity.

### Microarray and statistical analysis

MiRNA microarray (Affymetrix miRNA 4.0) was conducted by PREMEDICAL Co. Ltd. (Beijing, China). The microarray was utilized to find the aberrant expression of miRNAs from the POCD model mice to normal mice. Fluorescent signals were transformed from picture signal to digital data based on the degree of fluorescent for each probe, and then the data were saved as. DAT files by AGCC software (Affymetrix Genechip Command Console Software). The differential expression analyses were performed by using Transcriptome Analysis Console (v 4.0) and using an FDR correction for multiple testing. Considering that none of the identified miRNAs reached the threshold of adjusted significance, we chose the threshold of a nominal *P* value of < 0.05 for further replication.

The miRNA expression profile data of replication cohort were downloaded from the Gene Expression Omnibus (GEO) database (Accession number: GSE95070; https://www.ncbi.nlm.nih.gov/geo/query/acc.cgi?acc=GSE95070), which were deposited by Wei et al. [19] from the Chaoyang Hospital. With regard to this dataset, hippocampus tissues of 10 mice (5 per group) were dissected, and the different miRNA expression levels between two groups were detected by Affymetrix miRNA 4.0 as well. Similarly, the differential expression analysis was performed by using the Transcriptome Analysis Console (v 4.0) with same parameter.

### Quantitative real-time PCR

Expression level of the most significantly aberrant miRNA, mmu-miR-190a-3p, was validated by using real-time PCR assay. Reverse transcription reaction was performed with M-MLV Reverse Transcriptase kit (Takara Code: D2639A) based on the manufacturers’ protocol. Real-time PCR was performed with SYBR Premix Ex Taq kit (Takara Code: DRR041A). The miRNA expression level was evaluated relative to the expression of U6 of the 2 ^-ΔΔCt^. The primers for miRNA mmu-miR-190a-3p are listed in Additional file 1: Table S1.

### Statistical analysis

Data were analyzed using GraphPad PRISM (version 6; GraphPad Prism Software, Inc. San Diego, CA, USA). Measurements of dwelling time, number of grossing, escape latency, and speed in MWM test among preoperative and postoperative mice were analyzed by using Student’s t-test. qRT-PCR data were also analyzed with Student’s t-test. A *P* value of < 0.05 was considered statistically significant.

### WGCNA analysis

The genes and miRNAs expression data of GSE73507 were acquired from GEO database (https://www.ncbi.nlm.nih.gov/geo/query/acc.cgi?acc=GSE73507). The GSE73507 dataset was designed to gain insight into the relationship between the CAG repeat length and the Huntington disease [23]. By excluding the expression data from other brain regions and mutant mice, we only downloaded the mRNAs and miRNAs expression data from hippocampus tissue of wild type mice for our analysis (*n* = 24). Because of recent progress of alignment and mapping approaches, which are capable of detecting the transcriptome profiles in a more accurate and effective way compared with former tools [24]. The RNA and miRNA sequencing data were aligned and mapped to the GRCm38 version of mice genome using Hisat2 (v 2.1.0) and StringTie (v 1.3.4) [25], and the miRNA expression data by miRDeep2 (v 2.0.0.7) [26]. A total of 16,425 mRNAs and 1057 miRNAs were correctly mapped onto the mouse genome. Threshold for filtering out genes expressed at low levels was set to greater than 1 of the average fpkm. After the filtering process, 13,241 mRNAs and 546 miRNAs were included for WGCNA analysis.

The R package of WGCNA was used to construct the network modules of highly correlated transcripts subsets [27]. This approach aims to find the gene pairs with similar expression patterns and highly topological overlap, and it represents a valuable tool for identifying promising target genes and understanding the pathology of complex disorders [28]. In order to provide a comprehensive expression pattern among the mRNAs and miRNAs and detect the interaction of the transcripts, we performed our co-expression analysis by combined the mRNA and miRNA dataset together. First, we constructed a weighted network according to the gene pair correlations among all the mRNAs and miRNAs; second, by using the default parameters to assess the network interconnection, 25 specific modules were hierarchically clustered. These module sizes were from 50 to 17,500 genes. In the network we only showed a connection of the corresponding topological overlap is above a threshold of 0.05 with mmu-miR-190a-3p in the red module (*n* = 169). The visual gene-gene network plot was displayed by using the Cytoscape version 3.5.1 (https://www.cytoscape.org/) [29].

Concordance between the highly correlated mRNAs with mmu-miR-190a-3p and those not involved in the module genes were assayed by performing density plots. We compared the distribution of Pearson correlation coefficients of the 169 potential interaction targets of mmu-miR-190a-3p to a control distribution of non-predicted targets which consisted of all other mRNAs that we mapped. The mRNAs which were possibly modulated by mmu-miR-190a-3p displayed more significant negative correlation compared with the control.

### Pathway analysis

ClueGO (v. 2.3.4), a plug-in software of Cytoscape, was used to decipher the pathways network and determine their biological functions for the candidate genes [30]. The potential biological functions of each gene set were annotated using the pathway profiles of Kyoto Encyclopedia of Genes and Genomes (KEGG) [31].

### Target prediction

We conducted further analysis to screen the most potential regulated genes by mmu-miR-190a-3p. We first employed the miRWalk3.0 (http://mirwalk.umm.uni-heidelberg.de/) to predict the target genes regulated by mmu-miR-190a-3p [32]. By using a stringent standard to obtain reliable targets (Additional file 1: Table S2), we set the parameters for target prediction in the miRwalk3.0 as following: 1) Binding over than 0.9; 2) Energy less than − 16; and 3) Accessibility less than 0.05. Second, using these predicted targets for mmu-miR-190a-3p to overlap with the WGCNA results of those highly correlated genes. Finally, we conducted a protein-protein interaction network analysis for those overlapped genes according to the STRING v 10.5 under default parameters (https://string-db.org/cgi/input.pl). By combining the gene-gene interaction result, prediction targets and the protein-protein interaction analysis, we attempted to find the high degree receivable genes regulated by mmu-miR-190a-3p.

## Results

### Morris water maze test

Our MWM test showed that the dwelling time (*p* = 0.002) and the number of crossings (*p* = 0.049) of mice in Surgery group were significantly decreased than those in Sham group (Table 1). The swimming speed did not differ between the two groups (*p* = 0.660), however, the escape latency increased significantly in the Surgery group (*p <* 0.001). These results demonstrated that the lower ability to find the platform was not a result from the motor ability, but derived from the spatial learning memory impairment and the cognitive impairment. This indicates that the spatial learning ability of the mice in the Surgery group was impaired notably and a POCD mouse model has been developed.

**Table 1:** Results of Morris Water Maze test among preoperative and postoperative mice

Sample ID	Dwelling Time	Number of Crossing	Escape Latency	Speed
CTRL1	33.1	1.0	36.3	0.2
CTRL2	16.9	2.0	36.0	0.2
CTRL3	26.3	1.0	60.0	0.2
CTRL4	23.5	2.0	60.0	0.2
CTRL5	36.3	3.0	47.9	0.2
CTRL6	16.2	3.0	31.9	2.1
CTRL7	25.9	2.0	46.9	0.2
CTRL8	26.3	2.0	43.1	0.2
CASE1	26.0	2.0	60.0	0.3
CASE2	15.4	2.0	55.0	0.2
CASE3	13.7	1.0	60.0	0.2
CASE4	4.8	0.0	60.0	1.3
CASE5	30.6	2.0	60.0	0.2
CASE6	12.3	1.0	60.0	0.2
CASE7	17.1	1.0	56.3	0.2
CASE8	18.0	1.0	53.6	0.2
*p* Value	0.044	0.0596	0.005	0.754

Note: CTRL represents preoperative mice, CASE represents postoperative mice

### Differential expression analysis

**Fig. 1:**
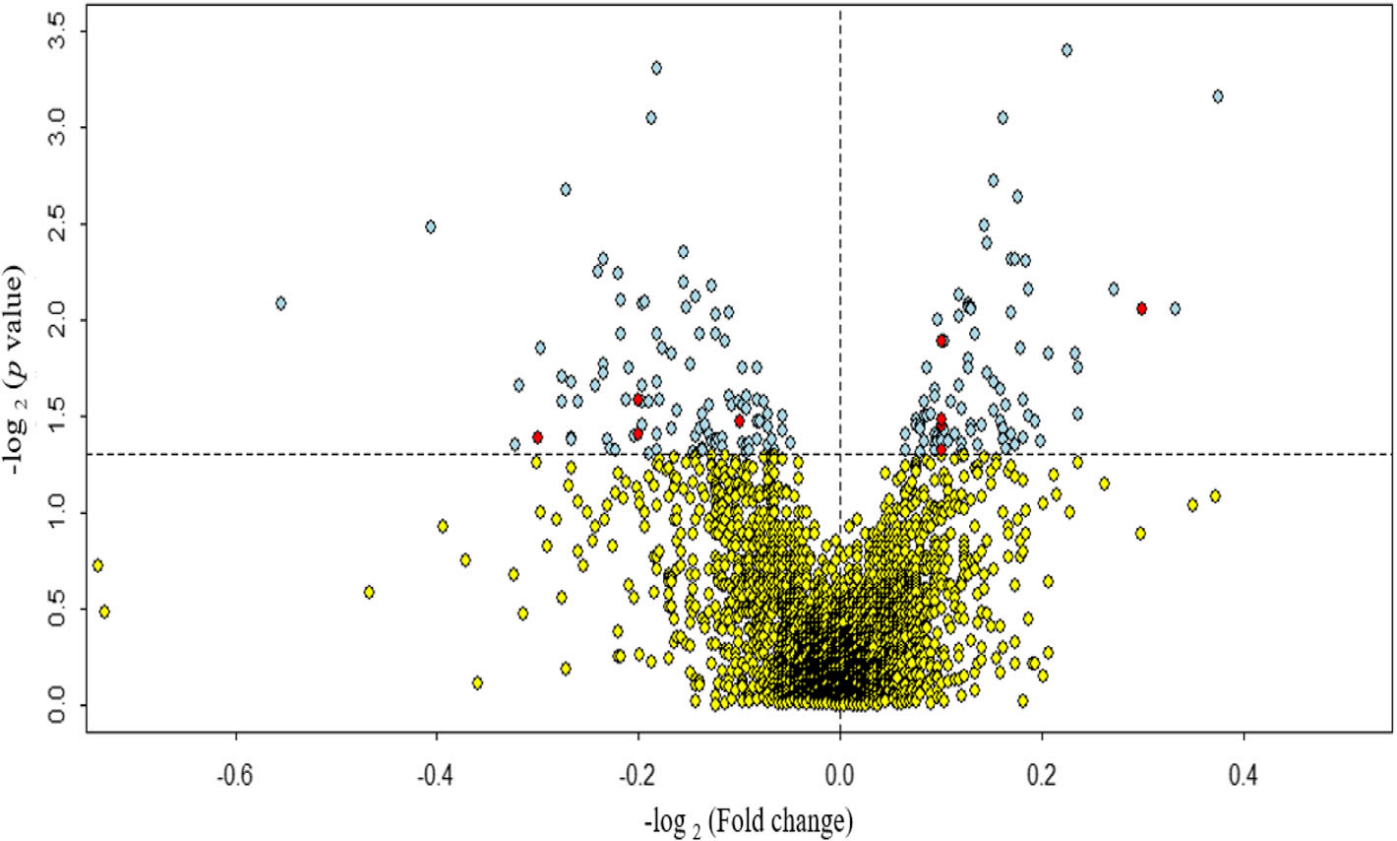
Volcano plot of miRNA expression in discovery cohort. Plotted along the x-axis is the mean of log2 fold-change, along the y-axis the negative logarithm of the log2 *p* values. Light blue demonstrates the miRNAs with significant *p* value (*p* < 0.05), and red denotes these significant miRNAs validated by the replication cohort. The horizontal line represents the threshold of significant *p* value

In order to assess the effects of aberrantly expressed miRNAs in hippocampus tissue of POCD mice brain, we performed differential miRNAs expression analysis of six samples. A total of 3162 mice miRNAs have been mapped. Compared with the mice in the Sham group, the microarray analysis results showed that 83 miRNAs were up-regulation and 103 miRNAs were down-regulation (*p* < 0.05) (Fig. 1).

Further, we performed a replication analysis with the same procedure by using a published miRNA microarray dataset (i.e., GSE95070 dataset). In this miRNAs expression profile, we detected 85 significantly up-regulation miRNAs and 103 significantly down-regulation miRNAs (*p* < 0.05) (Additional file 2: Figure S2). By comparing the analysis results from these two datasets, we found that five up-regulation miRNAs and four down-regulation miRNAs, which were significantly changed in both datasets with the same direction (Table 2).

**Table 2:** The replicated differential expressed miRNAs in hippocampus tissues

miRNA	Chr	Start	End	Discovery Cohort		Replication Cohort	
				Log2 (FC)	*p* Value	Log2 (FC)	*p* Value
mmu-miR-190a-3p	chr9	67,236,664	67,236,685	2.4	7.0E-04	1.9	2.4E-03
mmu-mir-7648	chr15	90,224,360	90,224,412	1.3	1.3E-02	1.1	2.3E-02
mmu-mir-1907	chr15	50,889,025	50,889,114	1.2	3.3E-02	1.4	2.1E-02
mmu-miR-184-5p	chr9	89,802,302	89,802,323	1.2	3.5E-02	1.2	3.9E-02
mmu-miR-6999-5p	chr2	91,944,908	91,944,930	1.2	4.8E-02	1.3	3.6E-02
mmu-miR-496a-3p	chr12	109,739,165	109,739,186	−1.5	2.6E-02	−1.1	3.3E-02
mmu-miR-592-3p	chr6	27,936,672	27,936,693	−1.9	4.1E-02	−2.3	6.6E-05
mmu-miR-6389	chr7	57,581,070	57,581,090	−1.5	3.9E-02	−1.2	5.0E-02
mmu-miR-6939-5p	chr12	112,659,327	112,659,347	−1.2	3.4E-02	−1.4	4.0E-02

Notes: 1) Chr: Chromosome; 2) FC: Fold Change

Among of these replicated targets, mmu-miR-190a-3p was the most significantly one in the Surgery group (i.e., POCD model mice) compared to the mice of the Sham group. For mmu-miR-190a-3p, it was significantly increased in the mice with POCD (Discovery sample: log2(FC) = 2.4, *p* = 7.0 × 10^− 4^; Replication sample: log2(FC) = 1.9, *p* = 2.4 × 10^− 3^). Real-time quantitative PCR was performed to technically validate the expression pattern of this miRNA in our discovery sample. Consistently, the result showed that the mmu-miR-190a-3p was significantly increased in the hippocampus tissue of mice with POCD (log2(FC) = 1.8, *p* = 0.041; see Additional file 2: Figure S3).

### Co-expression network analysis

**Fig. 2:**
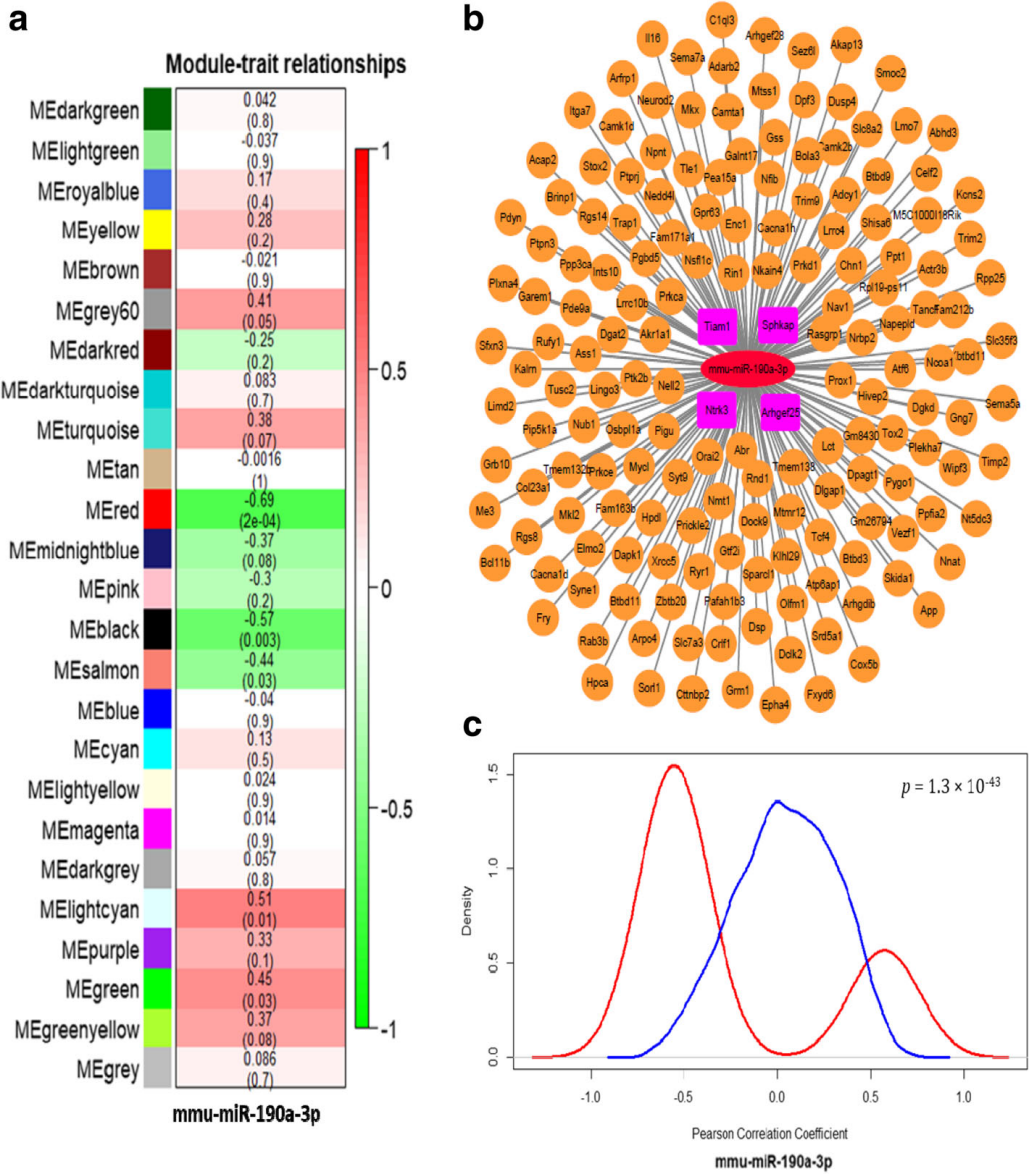
The mmu-miR-190a-3p involved module in the hippocampal tissue. (**a**) Clustering dendrograms of genes according to the topological overlap to distinguish the subgroup and used same color to assign highly correlated modules. As the result, 25 co-expression modules were constructed and painted with different colors. Pearson correlation analysis was used to determine whether co-expression modules are correlated with mmu-miR-190a-3p. Red color indicates the module was positively correlated with mmu-miR-190a-3p, and green demonstrates negative correlation. (**b**) Overview of candidate genes modified by the mmu-miR-190a-3p. Nodes are defined as the target genes modulated by the mmu-miR-190a-3p which was representing by red ellipse. The 4 pink rectangular represented the prediction results from miRwalk3.0 databases. (**c**) Distribution of Pearson correlation coefficients of predicted mmu-miR-190a-30-mRNA pairs. We determined the correlation between the expression of that miRNA and the expression of a predicted mRNA targets in all samples by calculating a Pearson correlation coefficient. This process was repeated for all targets and the red line indicated the distribution of these coefficients as a density plot. The blue line as the control curve represented the distribution of the coefficient of others unrelated mRNA with mmu-miR-190a-3p. A lift shift of the red line comparing with the red line demonstrated that the potential targets for mmu-miR-190a-3p are preferentially more negative correlated with targets gene than random

To determine the relevant POCD pathways modulated by target miRNAs, we investigated the orchestrating of the transcriptome in the hippocampus of mice brain by performing weighted gene co-expression network analysis to the 24 specimens as described in Materials and Methods. We detected 25 modules with highly significant co-expression patterns (Additional file 2: Figure S4). Because the mmu-miR-190a-3p was significantly highly expressed in the POCD model mice brain and one of the most significant miRNA in two samples (Table 2), we paid special attention on this miRNA in this communication. In addition, as showed in the Fig. 2a, mmu-miR-190a-3p was significantly inverse-correlated with the red module.

**Fig. 3:**
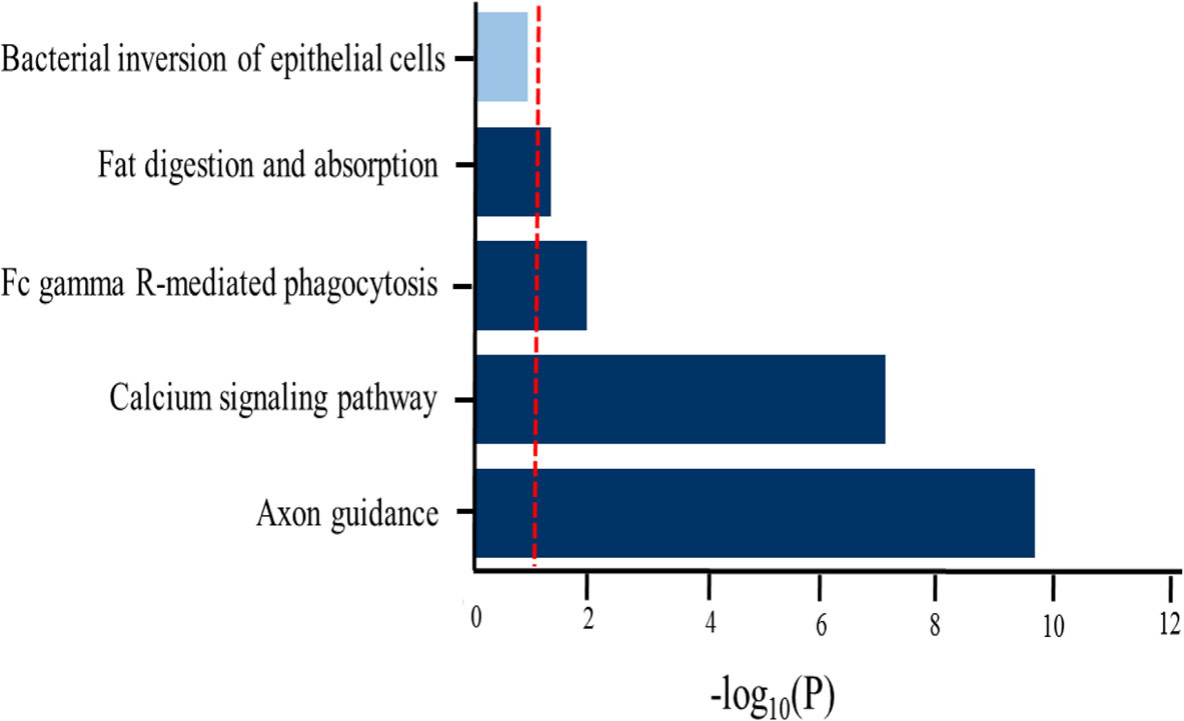
KEGG pathway enrichment analysis for genes in red module. The red vertical line indicates the threshold of the significant pathway enrichment *p* value after FDR correction. Dark blue color of horizontal bars represents the significantly enriched pathway, and the light blue color represents the non-significantly enriched pathway

A total of 314 genes and 5 miRNAs were grouped in the red module (Additional file 1: Table S3). Further, by conducting the pathway analysis of the genes in red module, we found four significant enrichment pathways (Fig. 3). The most significant pathway was axon guidance (*p* = 1.9 × 10^− 10^), and the second to fourth pathways were calcium signaling pathway (*p* = 7.7 × 10^− 08^), Fc gamma R-mediated phagocytosis (*p* = 1.2 × 10^− 2^), and Fat digestion and absorption (*p* = 5.0 × 10^− 02^), respectively. Among these four significant pathways, the axon guidance, calcium signaling pathway and Fc gamma R-mediated phagocytosis have been widely identified to be related with the psychiatric disorders and brain development [33–38].

In addition, we refined 169 genes from red module with a highly connected with mmu-miR-190a-3p (Fig. 2b). To determine whether these highly correlated genes have critical roles in psychiatric disorders, we performed similar pathway-based enrichment analysis of these 169 genes and found several significant enriched pathways (Additional file 2: Figure S5), which was in line with the results based on all genes in red module. These findings indicated that the mmu-miR-190a-3p acted as a key regulator to red module genes, which might be involved in the pathology of psychiatry diseases. Since miRNAs negatively altered the expression of its target genes [39], we expect a left shift in the predicted target genes distribution toward the negative correlations compared with the background. To explore this possibility, we compared the Pearson correlation coefficients for mmu-miR-190a-3p with its predicted targets. Indeed, we found a significantly left shift of these target genes compared with randomly selected non-predicted targets (*p* = 1.3 × 10^− 43^; Fig. 2c).

**Fig. 4:**
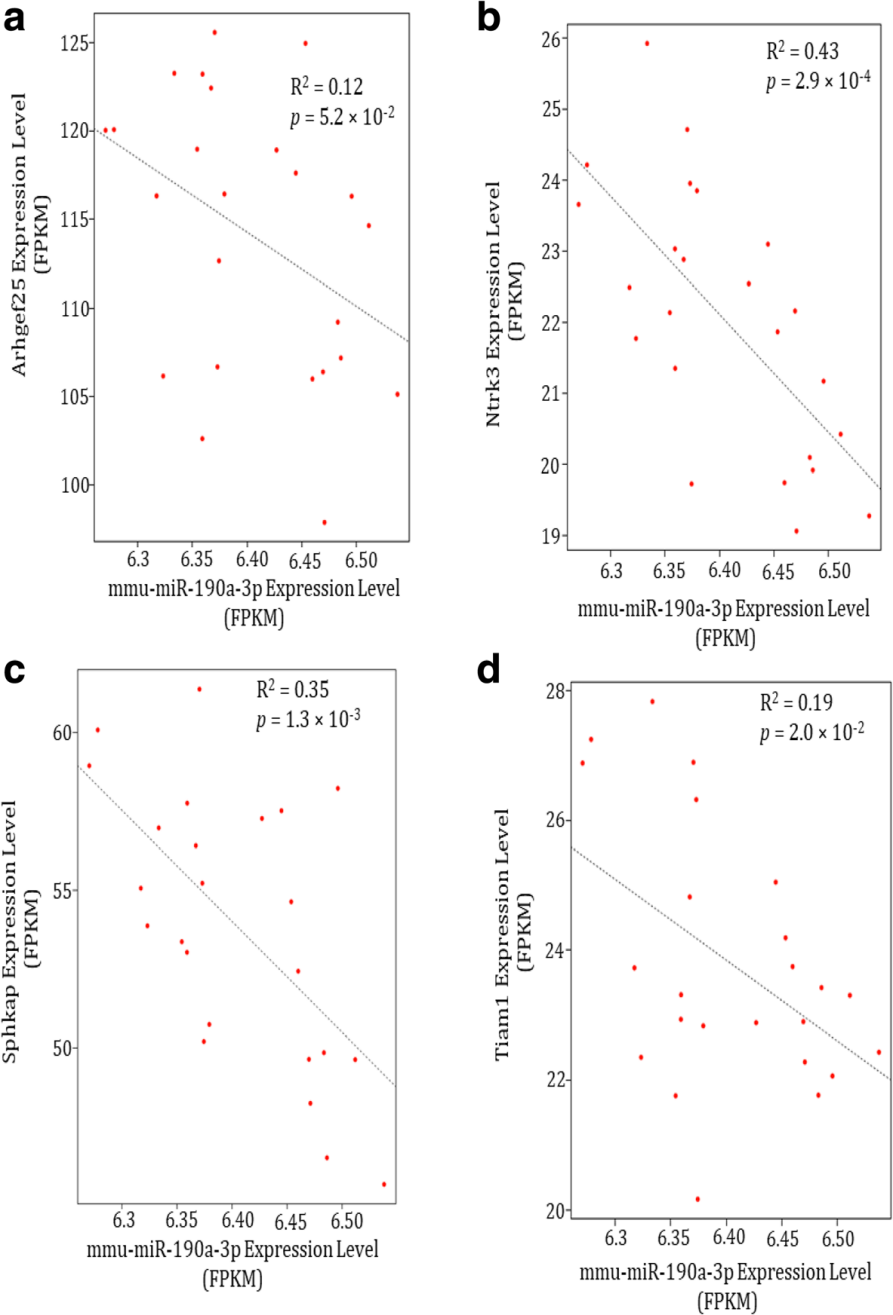
Prediction binding targets show anti-correlation with mmu-miR-190a-3p. The target mRNAs expression level (FPKM) of: **a**) *Arhgef25*; **b**) *Ntrk3*; **c**) *Sphkap* and **d**) *Tiam1* are inversely correlated with the expression level of mmu-miR-190a-3p

**Fig. 5:**
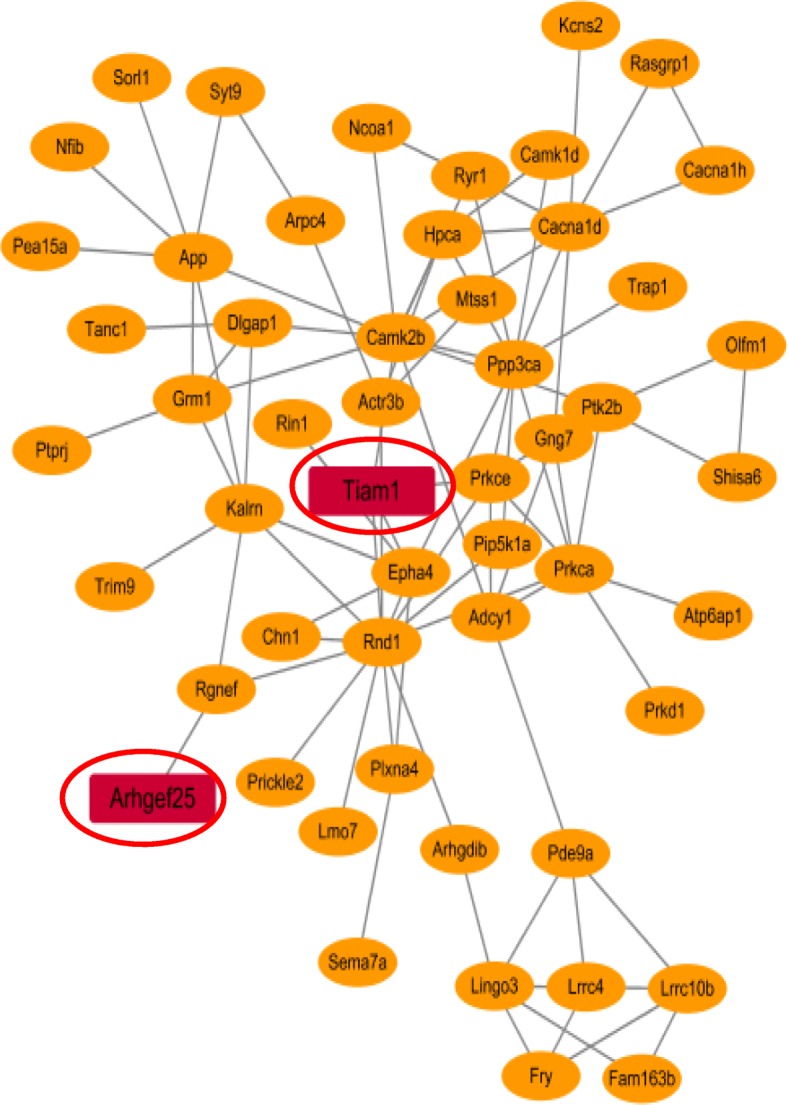
Protein-protein interaction network for mmu-miR-190a-3p potential regulated genes in mice hippocampus tissue. The genes overlapped with prediction targets by mmu-miR-190a-3p were highlighted as red rectangular

As a result, we found that 4 of 169 correlated genes (*Sphkap*, *Arhgef25*, *Tiam1*, and *Ntrk3*) involved putative binding sites at 3′-UTR and CDS regions of mmu-miR-190a-3p (Additional file 1: Table S2). Further, we performed correlation analysis with mmu-miR-190a-3p and 4 targets genes. As shown in Fig. 4, the mmu-miR-190a-3p was significantly negative correlated with the expression levels of *Sphkap* (R^2^ = 0.35, *p* = 1.3 × 10^− 3^), *Tiam1*(R^2^ = 0.19, *p* = 2.03 × 10^− 2^) and *Ntrk3* (R^2^ = 0.43, *p* = 2.9 × 10^− 4^), respectively. As for *Arhgef25*, we observed a marginally significant correlation with the mmu-miR-190a-3p (R^2^ = 0.12, *p* = 5.2 × 10^− 2^). Importantly, as showed in Fig. 5, the evidence from protein-protein interaction analysis displayed that *Tiam1* as a hub gene was possibly dysregulated by mmu-miR-190a-3p.

## Discussion

In this study, we examined dysregulated miRNAs in the hippocampus of POCD mice and further analyzed the potential mechanisms of the target miRNAs in this neurocognitive disorder. Firstly, based on two independent miRNA microarray datasets, we identified 9 significant dysregulated miRNAs in the hippocampus tissues of POCD mice. Among of these identified miRNAs, mmu-miR-190a-3p was the most significantly differential expressed one in both datasets, and this had been validated by using the qRT-PCR. Secondly, we used a sequence-based dataset of both mRNA and miRNA data to perform co-expression analysis, which revealed 25 co-expression modules. Interestingly, the co-expression red module including mmu-miR-190a-3p was of significant enrichment in the neuron-related pathways and immunology pathways, which have been well-documented to play crucial roles in the psychiatry diseases [33–38]. Finally, by using the database of miRwalk3.0 to conduct miRNA targets prediction analysis, we found *Tiam1*, a Rac-specific guanine nucleotide exchange factor (GEF) required high level expression in development of spine and synapse, might be repressed by mmu-miR-190a-3p in the POCD patients.

Previous studies have reported that several miRNAs were significantly associated with POCD [6, 18–20], which provide supportive evidence of the important role of miRNAs in POCD. Consistently, some of those reported miRNAs were replicated in our current study. For example, Chen and colleagues [20] reported that mmu-miR-146a showed increased expression in the BV-2 microglial cells stimulated with LPS and hippocampal tissues of mice with POCD. For the mmu-miR-146a, we observed that it was marginally significantly associated with POCD (Log2 (FC) = 1.1; *P* = 0.054). In addition, Shan et al. illuminated that the expression of mmu-miR-30a was significantly decreased in the mPFC and hippocampus of aged rats compared with control and was involved in the expression regulation of several innate immune system related genes [40]. Such a finding was validated by our study, where we found that the mmu-miR-30a decreased the expression level in the POCD model mice compared with control (Log2 (FC) = − 1.2; *P* = 0.046).

Furthermore, we also performed an integrative analysis to refine a collection of putative mRNA-miRNA interaction pairs for mmu-miR-190a-3p. By applying to a powerful approach, which was combining expression data of miRNAs and mRNAs while performing the co-expression analysis based on Pearson correlation, we provided a new clue to detect the biological network relationship between miRNA and mRNA [41–44]. Because the primary function of miRNAs was to inhibit expression of target genes [39], it was reasonable to consider that there were more significantly negative correlation coefficient between mmu-miR-190a-3p and its target genes than the background genes. Consistently, we found that the target genes of mmu-miR-190a-3p were significantly shifted to negative part whereas the background genes were always presented a normal distribution. According to the prediction results from miRwalk3.0, we filtered out the genes with no binding sites to mmu-miR-190a-3p, which lead to the identification of likely four target genes for mmu-miR-190a-3p. Also, based on the protein-protein interaction analysis, two of four putative genes, *Tiam1* and *Arhgef25*, were involved in the interaction network. Interestingly, *Tiam1,* as a hub gene located on the central of network map, might be modified by abnormal expression of mmu-miR-190a-3p and played important roles in the pathogenesis of POCD.

The majority of excitatory synapses existed in micron-sized dendritic protrusions of the mammalian brain, which can change along with the variation of synaptic activity in terms of the alternation in number and shape [45]. *Tiam1* is widely expressed in the developing central nervous system [46]. *Tiam1* is one of the important Rac-specific GEFs which coordinate activation of the GTPase Rac1 with other GEFs in many partitioning-defective (PAR) processes within spines [47]. The PAR complex is an essential determinant of cellular polarity [48, 49]. This complex included Par3, Par6, and atypical protein kinase C, which modulates polarized processes, like axon specification, neuronal migration, and development of spine and synapse [50–52]. Par3 combines with Tiam1 together to exert vital effects on cellular polarity by spatially restricting Rac1’s activation and localization in cyto-skeletal remodeling [47]. Due the loss of Tiam1’s function in synapses, the Rac1’s activation and spinogenesis cannot be formed in neurons from glutamate/NMDA receptor signaling and ephrinB/EphB receptor signaling [53, 54]. If the Par3 were knock-down and Tiam1 were delocalized in the neurons, it would result in Rac1’s dysregulation and synaptic digenesis [52]. In addition, *Tiam1* activates and recruits to the membranes after exposure to amyloid beta peptide in hippocampal neurons, and this might exert influence to the pathology of Alzheimer disease [55, 56]. A study reported that *Tiam1* knockdown declined hippocampal neuronal vulnerability to oxygen/glucose depravation [57]. Conclusively, these results demonstrated that the *Tiam1* is an important gene for the hippocampal neurons’ development, and for the etiology of several psychiatry diseases. We showed here that increasing expression levels of mmu-miR-190a-3p might inhibit the activation of the Tiam1/Rac1 pathway in the hippocampus after surgery.

There were a few of limitations in this study. First, due to the limited number of sample size and low expression level of miRNA in brain, some weak signals of miRNAs were difficulty to be observed. To obtain more reliable expression signals, we used both the public data from GEO database and RT-PCR to validate the significantly differential expressed miRNAs. Second, although we employed various robust bioinformatics methods to find the binding targets of mmu-miR-190a-3p, such as the expression of *Trim1*gene which may be restrained by mmu-miR-190a-3p in the hippocampus of POCD, we did not confirm these gene-miRNA interactions experimentally, which should be the research focus of future study.

## Conclusions

To sum up, we provided robust evidence supporting that mmu-miR-190a-3p has a strong positive correlation with the incidence of POCD. The pathway analysis displayed that these mmu-miR-190a-3p targeted genes were enriched in the psychiatry-related pathways and immune system-related pathways. Among of these target genes, *Tiam1* was inhibited by mmu-miR-190a-3p in the hippocampus tissue of POCD model mice. These results demonstrated that mmu-miR190a-3p probably serve as an important regulator to inhibit target genes expression in the hippocampus tissue of POCD patients. More molecular-based studies are warranted to explore the underlying biological mechanisms of target genes by mmu-miR-190a-3p in the pathogenesis of POCD.

## Additional files

Additional file 1: **Table S1.** The primer sequence of the validated miRNA. i.e., the primer sequence of mmu-miR-190a-3p used for real-time PCR assay. **Table S2.** Predictionbinding sites for mmu-miR-190a-3p. The data were predicted by using miRwalk 3.0 for obtaining reliable targets for mmu-miR-190a-3p. **Table S3.** Genes and miRNAs involved in red module. This red module were generated by using our co-expression weighted network analysis by combined the 314 mRNA and 5 miRNAs data. (DOCX 49 kb)

Additional file 2: **Figure S1.** The detailed time line of the euthanization of study animals. According to the protocol, all mice were euthanized before obtaining hippocampal tissue. All mice were deeply anesthetized based on several signs (see Methods part). Then the mice were decapitated 24 h after operation, brains were instantly dissected on ice, and the hippocampal tissues were obtained and stored in liquid nitrogen. **Figure S2.** Volcano plot of miRNA expression in replication cohort. This dataset (GSE95070) was downloaded from the GEO database. **Figure S3.** The results of qRT-PCR for technical replication. Expression level of the most significantly mmu-miR-190a-3p was validated by using real-time PCR assay. Reverse transcription reaction was performed with M-MLV Reverse Transcriptase kit (Takara Code: D2639A) based on the manufacturers’ protocol. **Figure S4.** WGCNA module-based analysis for genes and miRNAs expression data. The genes and miRNAs expression data of GSE73507 were acquired from GEO database. After the filtering process, 13,241 mRNAs and 546 miRNAs from hippocampus tissue of wild type mice (*n* = 24) were included for WGCNA analysis. **Figure S5.** KEGG pathway analyses for mmu-miR-190a-3p highly related genes in red module. We refined 169 genes from red module with a highly connected with mmu-miR-190a-3p (Fig. 2b) and used ClueGO (v. 2.3.4) to decipher the pathways and determine their biological functions. (PPTX 118 kb)

## Data Availability

The datasets generated and/or analysed during the current investigation are available from the corresponding author until it becomes available from a public repository.

## Abbreviations

AGCC: Affymetrix Genechip Command Console
circRNA: circular RNA
GEO: Gene expression omnibus
KEGG: Kyoto Encyclopedia of Genes and Genomes
lncRNA: long non-coding RNA
miRNA: microRNA
MWM: Morris water maze
PCR: Polymerase chain reaction
POCD: Postoperative cognitive dysfunction
WGCNA: Weighted gene co-expression network analysis
