# Supplementary material for: MiR-190a potentially ameliorates postoperative cognitive dysfunction by regulating Tiam1
Source: BMC Genomics. 2019 Aug 22;20:670. doi: 10.1186/s12864-019-6035-0 (PMC6704709; doi:10.1186/s12864-019-6035-0)
Supplement: Supplementary file 1 — Table S1. The primer sequence of the validated miRNA. i.e., the primer sequence of mmu-miR-190a-3p used for real-time PCR assay. Table S2. Predictionbinding sites for mmu-miR-190a-3p. The data were predicted by using miRwalk 3.0 for obtaining reliable targets for mmu-miR-190a-3p. Table S3. Genes and miRNAs involved in red module. This red module were generated by using our co-expression weighted network analysis by combined the 314 mRNA and 5 miRNAs data. (DOCX 49 kb) [file 12864_2019_6035_MOESM1_ESM.docx]

**Additional file 1: Table S1. Primer Sequence of the validated miRNA**

| miRNA ID | MiRbase accession No. | Sequence |
| --- | --- | --- |
| mmu-miR-190a-3p | MIMAT0016998 | acuauauaucaagcauauuccu |

**Additional file 1: Table S2. Prediction binding sites for mmu-miR-190a-3p from miRwalk 3.0**

| miRNA ID | Gene ID | Start | End | Bindingp | Energy | Accessibility | Binding Position |
| --- | --- | --- | --- | --- | --- | --- | --- |
| mmu-miR-190a-3p | Sphkap | 5914 | 5936 | 0.92 | -16.3 | 4.1E-03 | 3UTR |
|  | Arhgef25 | 1233 | 1255 | 0.92 | -17.8 | 2.5E-04 | CDS |
|  | Tiam1 | 3735 | 3753 | 0.92 | -16.7 | 1.6E-02 | CDS |
|  | Tiam1 | 633 | 651 | 0.92 | -16.7 | 1.6E-02 | CDS |
|  | Ntrk3 | 14763 | 14787 | 1.00 | -22.2 | 2.2E-02 | 3UTR |
|  | Ntrk3 | 14401 | 14425 | 1.00 | -22.2 | 2.2E-02 | 3UTR |

**Additional file 1: Table S3. Genes and miRNAs involved in red module**

| Gene_id | Chr | Strand | Start position | End position | Genes correlated with mmu-miR-190a-3p  (Weight > 0.05) |
| --- | --- | --- | --- | --- | --- |
| *Rrs1* | 1 | + | 9545408 | 9547455 | No |
| *Jph1* | 1 | - | 16964560 | 17097889 | No |
| *Cox5b* | 1 | + | 36691484 | 36693681 | Yes |
| *Tmem131* | 1 | - | 36792191 | 36943666 | No |
| *2010300C02Rik* | 1 | - | 37611677 | 37720085 | No |
| *Slc9a2* | 1 | + | 40680574 | 40769273 | No |
| *Als2* | 1 | - | 59162926 | 59237231 | No |
| *Xrcc5* | 1 | + | 72307421 | 72394952 | Yes |
| *Epha4* | 1 | - | 77367185 | 77515088 | Yes |
| *Dock10* | 1 | - | 80501073 | 80758527 | No |
| *Sphkap* | 1 | - | 83254139 | 83408200 | Yes |
| *Dgkd* | 1 | + | 87853287 | 87945180 | Yes |
| *Lct* | 1 | - | 128284756 | 128328318 | Yes |
| *Mcm6* | 1 | - | 128331590 | 128359664 | No |
| *Mfsd4a* | 1 | - | 132022806 | 132068062 | No |
| *Arl8a* | 1 | + | 135146824 | 135156269 | No |
| *Nav1* | 1 | - | 135434580 | 135607295 | Yes |
| *Rgs8* | 1 | + | 153653025 | 153700323 | Yes |
| *Rnasel* | 1 | + | 153749426 | 153764221 | No |
| *2810025M15Rik* | 1 | + | 157412352 | 157420236 | No |
| *Rxrg* | 1 | + | 167598384 | 167639623 | No |
| *Atf6* | 1 | - | 170704674 | 170867771 | Yes |
| *Pea15a* | 1 | - | 172196728 | 172206804 | Yes |
| *Parp1* | 1 | + | 180568924 | 180601254 | No |
| *Prox1* | 1 | - | 190118035 | 190170714 | Yes |
| *Gm38259* | 1 | - | 8527780 | 8531313 | No |
| *Rp1* | 1 | - | 3999557 | 4409241 | No |
| *Fam171a1* | 2 | + | 3114224 | 3227806 | Yes |
| *Optn* | 2 | - | 5020642 | 5064051 | No |
| *Camk1d* | 2 | - | 5293457 | 5714515 | Yes |
| *Celf2* | 2 | - | 6539694 | 7509563 | Yes |
| *C1ql3* | 2 | - | 13003457 | 13011806 | Yes |
| *Skida1* | 2 | - | 18040676 | 18049051 | Yes |
| *Fam163b* | 2 | - | 27110380 | 27142491 | Yes |
| *Olfm1* | 2 | + | 28192992 | 28230736 | Yes |
| *Ass1* | 2 | + | 31470207 | 31520672 | Yes |
| *Pbx3* | 2 | - | 34171457 | 34373142 | No |
| *Gm26794* | 2 | - | 55433514 | 55437860 | Yes |
| *Acvr1* | 2 | - | 58388644 | 58567157 | No |
| *Tanc1* | 2 | + | 59612042 | 59846149 | Yes |
| *Chn1* | 2 | - | 73610660 | 73775346 | Yes |
| *Ptprj* | 2 | - | 90429754 | 90580647 | Yes |
| *Arhgap1* | 2 | + | 91649860 | 91672326 | No |
| *Mpped2* | 2 | + | 106693269 | 106868356 | No |
| *Rasgrp1* | 2 | - | 117279993 | 117343001 | Yes |
| *Gpr176* | 2 | - | 118277110 | 118373419 | No |
| *Rhov* | 2 | - | 119269201 | 119271272 | No |
| *Pdyn* | 2 | - | 129686565 | 129699844 | Yes |
| *Chgb* | 2 | + | 132781278 | 132795079 | No |
| *Btbd3* | 2 | + | 138256565 | 138589292 | Yes |
| *Ralgapa2* | 2 | - | 146239879 | 146512344 | No |
| *Syndig1* | 2 | + | 149829211 | 150004392 | No |
| *Nsfl1c* | 2 | + | 151494182 | 151511414 | Yes |
| *Tspyl3* | 2 | - | 153222370 | 153225441 | No |
| *Pigu* | 2 | - | 155278243 | 155357430 | Yes |
| *Gss* | 2 | - | 155563181 | 155592810 | Yes |
| *Nnat* | 2 | + | 157560078 | 157562522 | Yes |
| *Tox2* | 2 | + | 163203125 | 163324170 | Yes |
| *Elmo2* | 2 | - | 165288031 | 165326479 | Yes |
| *Nkain4* | 2 | - | 180934772 | 180954699 | Yes |
| *Arfrp1* | 2 | - | 181357690 | 181365404 | Yes |
| *Oprl1* | 2 | + | 181715016 | 181720985 | No |
| *Mrps28* | 3 | - | 8802146 | 8923918 | No |
| *Trim2* | 3 | - | 84160439 | 84306877 | Yes |
| *Dclk2* | 3 | - | 86786151 | 86920852 | Yes |
| *Pip5k1a* | 3 | - | 95058530 | 95106930 | Yes |
| *Hist2h3c2* | 3 | - | 96238108 | 96239127 | No |
| *Fam212b* | 3 | + | 105704599 | 105720842 | Yes |
| *Npnt* | 3 | - | 132881745 | 132950291 | Yes |
| *Ppp3ca* | 3 | + | 136670124 | 136937727 | Yes |
| *Gpr63* | 4 | + | 24966407 | 25009233 | Yes |
| *Slc25a51* | 4 | - | 45395923 | 45408766 | No |
| *Zfp189* | 4 | + | 49521176 | 49531517 | No |
| *Ptpn3* | 4 | - | 57190841 | 57301837 | Yes |
| *Brinp1* | 4 | - | 68761514 | 68954397 | Yes |
| *Tle1* | 4 | - | 72117142 | 72200919 | Yes |
| *Dmac1* | 4 | - | 75277354 | 75278305 | No |
| *Nfib* | 4 | - | 82290173 | 82705750 | Yes |
| *Plpp3* | 4 | + | 105157347 | 105232764 | No |
| *Rab3b* | 4 | + | 108879063 | 108943324 | Yes |
| *Agbl4* | 4 | + | 110397661 | 111664324 | No |
| *Akr1a1* | 4 | - | 116636510 | 116651680 | Yes |
| *Hpdl* | 4 | - | 116819904 | 116821707 | Yes |
| *Ppt1* | 4 | + | 122836242 | 122859175 | Yes |
| *Mycl* | 4 | + | 122995652 | 123002485 | Yes |
| *Maneal* | 4 | - | 124855239 | 124862171 | No |
| *Hpca* | 4 | - | 129111570 | 129122036 | Yes |
| *Hdac1* | 4 | - | 129516104 | 129542713 | No |
| *Pla2g2f* | 4 | - | 138750533 | 138757626 | No |
| *Kazn* | 4 | - | 142102390 | 142239401 | No |
| *Camta1* | 4 | - | 150917322 | 151861876 | Yes |
| *Napepld* | 5 | - | 21662901 | 21701396 | Yes |
| *Atg9b* | 5 | - | 24384181 | 24392143 | No |
| *Nub1* | 5 | + | 24685532 | 24710378 | Yes |
| *Actr3b* | 5 | + | 25759997 | 25850688 | Yes |
| *Tyms* | 5 | - | 30058202 | 30073617 | No |
| *Babam2* | 5 | + | 31697684 | 32084962 | No |
| *Bloc1s4* | 5 | - | 36747378 | 36748650 | No |
| *Drd5* | 5 | + | 38319367 | 38322518 | No |
| *Ppargc1a* | 5 | - | 51454250 | 51567726 | No |
| *Adamts3* | 5 | - | 89677087 | 89883334 | No |
| *Sparcl1* | 5 | - | 104079111 | 104113733 | Yes |
| *Sez6l* | 5 | - | 112419151 | 112577185 | Yes |
| *Hrk* | 5 | + | 118164648 | 118189478 | No |
| *Tmem132b* | 5 | + | 125531774 | 125792583 | Yes |
| *Galnt17* | 5 | - | 130872082 | 131308497 | Yes |
| *Gtf2i* | 5 | - | 134237834 | 134314760 | Yes |
| *Orai2* | 5 | - | 136147459 | 136170713 | Yes |
| *Nptx2* | 5 | + | 144545902 | 144557478 | No |
| *Fry* | 5 | + | 150118645 | 150497753 | Yes |
| *Cttnbp2* | 6 | - | 18366478 | 18514843 | Yes |
| *Grm8* | 6 | - | 27275119 | 28135178 | No |
| *Lrrc4* | 6 | - | 28661831 | 28831747 | Yes |
| *Plxna4* | 6 | - | 32144268 | 32588192 | Yes |
| *Wipf3* | 6 | + | 54429603 | 54503768 | Yes |
| *Pde1c* | 6 | - | 56069804 | 56569103 | No |
| *Lrrtm4* | 6 | + | 80018877 | 80810143 | No |
| *Bola3* | 6 | + | 83349147 | 83360136 | Yes |
| *Cyp26b1* | 6 | - | 84571414 | 84593908 | No |
| *Prickle2* | 6 | - | 92370908 | 92706155 | Yes |
| *Arpc4* | 6 | + | 113378115 | 113390448 | Yes |
| *Slc6a1* | 6 | + | 114282635 | 114317532 | No |
| *Gm8430* | 6 | + | 122456904 | 122457374 | Yes |
| *Emg1* | 6 | - | 124704085 | 124712178 | No |
| *Smco3* | 6 | - | 136829927 | 136835452 | No |
| *Arhgdib* | 6 | - | 136923655 | 136941899 | Yes |
| *Slc8a2* | 7 | + | 16129826 | 16161063 | Yes |
| *Psg16* | 7 | + | 17074040 | 17133450 | No |
| *Pafah1b3* | 7 | - | 25295049 | 25297986 | Yes |
| *Ryr1* | 7 | - | 29003344 | 29125179 | Yes |
| *Akap13* | 7 | + | 75455534 | 75754609 | Yes |
| *Ntrk3* | 7 | - | 78175959 | 78738012 | Yes |
| *Il16* | 7 | - | 83642825 | 83745726 | Yes |
| *Gm2115* | 7 | + | 84528954 | 84583531 | No |
| *Me3* | 7 | + | 89632392 | 89854359 | Yes |
| *Dgat2* | 7 | - | 99153658 | 99182719 | Yes |
| *Fam168a* | 7 | + | 100706635 | 100841656 | No |
| *Syt9* | 7 | + | 107370728 | 107548656 | Yes |
| *Plekha7* | 7 | - | 116123485 | 116308376 | Yes |
| *Cend1* | 7 | - | 141426446 | 141429491 | No |
| *Mrgpre* | 7 | - | 143778363 | 143784500 | No |
| *Cttn* | 7 | - | 144435733 | 144471009 | No |
| *Efnb2* | 8 | - | 8617434 | 8661242 | No |
| *Ing1* | 8 | + | 11555571 | 11563251 | No |
| *Kbtbd11* | 8 | + | 15011025 | 15033333 | Yes |
| *Rpl19-ps11* | 8 | + | 19492936 | 19493526 | Yes |
| *Plekha2* | 8 | - | 25039144 | 25102376 | No |
| *Wrn* | 8 | - | 33234384 | 33385527 | No |
| *Dusp4* | 8 | + | 34807297 | 34819894 | Yes |
| *Stox2* | 8 | - | 47180048 | 47446362 | Yes |
| *Ints10* | 8 | + | 68793929 | 68831667 | Yes |
| *Crlf1* | 8 | + | 70493158 | 70504081 | Yes |
| *Mcm5* | 8 | + | 75109569 | 75128439 | No |
| *Mylk3* | 8 | - | 85324303 | 85386345 | No |
| *Tox3* | 8 | - | 90247040 | 90348343 | No |
| *Cpne2* | 8 | + | 94532990 | 94570531 | No |
| *1700030J22Rik* | 8 | - | 116969594 | 116978959 | No |
| *Pgbd5* | 8 | - | 124369049 | 124439658 | Yes |
| *Egln1* | 8 | - | 124908587 | 124949324 | No |
| *Slc35f3* | 8 | + | 126298558 | 126395482 | Yes |
| *Trpc6* | 9 | + | 8544142 | 8680741 | No |
| *Robo3* | 9 | - | 37415669 | 37433246 | No |
| *Sorl1* | 9 | - | 41964720 | 42124297 | Yes |
| *Dpagt1* | 9 | + | 44326019 | 44333900 | Yes |
| *Fxyd6* | 9 | + | 45370185 | 45396159 | Yes |
| *Rpp25* | 9 | + | 57504026 | 57505448 | Yes |
| *Sema7a* | 9 | + | 57940112 | 57962865 | Yes |
| *Gm17322* | 9 | + | 57998041 | 58007451 | No |
| *1600029O15Rik* | 9 | + | 58202897 | 58208808 | No |
| *M5C1000I18Rik* | 9 | - | 67758712 | 67760230 | Yes |
| *Pygo1* | 9 | + | 72925645 | 72952181 | Yes |
| *Tpbg* | 9 | + | 85842380 | 85847040 | No |
| *Rwdd2a* | 9 | + | 86571991 | 86574899 | No |
| *Il20rb* | 9 | - | 100457719 | 100486788 | No |
| *Rad54l2* | 9 | - | 106688082 | 106789194 | No |
| *Tusc2* | 9 | + | 107563255 | 107566112 | Yes |
| *Nicn1* | 9 | + | 108290429 | 108296498 | No |
| *Wdr6* | 9 | - | 108572311 | 108578739 | No |
| *Syne1* | 10 | - | 5020917 | 5551482 | Yes |
| *Grm1* | 10 | - | 10686059 | 11082356 | Yes |
| *Plagl1* | 10 | + | 13060504 | 13131694 | No |
| *Hivep2* | 10 | + | 13966075 | 14151374 | Yes |
| *Nmbr* | 10 | + | 14705591 | 14770850 | No |
| *Nhsl1* | 10 | + | 18318985 | 18533892 | No |
| *Lingo3* | 10 | - | 80832801 | 80844039 | Yes |
| *Gng7* | 10 | - | 80948624 | 81014945 | Yes |
| *Btbd11* | 10 | + | 85386814 | 85660292 | Yes |
| *Nt5dc3* | 10 | + | 86779005 | 86838389 | Yes |
| *Ppfia2* | 10 | + | 106470339 | 106935952 | Yes |
| *Arhgef25* | 10 | - | 127182525 | 127190083 | Yes |
| *Itga7* | 10 | + | 128933818 | 128958282 | Yes |
| *Camk2b* | 11 | - | 5969644 | 6066362 | Yes |
| *Ppia* | 11 | + | 6415443 | 6419817 | No |
| *Adcy1* | 11 | + | 7063489 | 7178506 | Yes |
| *Igfbp3* | 11 | - | 7206086 | 7213923 | No |
| *Grb10* | 11 | - | 11930508 | 12038683 | Yes |
| *Rufy1* | 11 | - | 50389286 | 50431125 | Yes |
| *Col23a1* | 11 | + | 51289920 | 51583918 | Yes |
| *Hist3h2ba* | 11 | + | 58948920 | 58949533 | No |
| *B9d1* | 11 | + | 61505144 | 61512931 | No |
| *Shisa6* | 11 | - | 66211725 | 66525964 | Yes |
| *Dhx33* | 11 | - | 70984091 | 71004437 | No |
| *Abr* | 11 | - | 76416734 | 76622314 | Yes |
| *Vezf1* | 11 | + | 88068279 | 88084729 | Yes |
| *Neurod2* | 11 | - | 98325415 | 98329648 | Yes |
| *Hap1* | 11 | - | 100347327 | 100356128 | No |
| *Nmt1* | 11 | + | 103028190 | 103068912 | Yes |
| *Limd2* | 11 | - | 106156256 | 106160860 | Yes |
| *Prkca* | 11 | - | 107933387 | 108343928 | Yes |
| *Kcnj16* | 11 | + | 110968033 | 111027968 | No |
| *Timp2* | 11 | - | 118301069 | 118355740 | Yes |
| *Ncoa1* | 12 | - | 4247362 | 4477182 | Yes |
| *Klhl29* | 12 | - | 5077472 | 5375682 | Yes |
| *Fam84a* | 12 | - | 14146830 | 14152054 | No |
| *Prkd1* | 12 | - | 50341231 | 50649098 | Yes |
| *Trim9* | 12 | - | 70244533 | 70347614 | Yes |
| *Dpf3* | 12 | - | 83213745 | 83487716 | Yes |
| *Acot5* | 12 | + | 84069325 | 84076020 | No |
| *Bcl11b* | 12 | - | 107910403 | 108003602 | Yes |
| *Meg3* | 12 | + | 109541001 | 109571726 | No |
| *Net1* | 13 | - | 3882018 | 3918220 | No |
| *Adarb2* | 13 | + | 8202866 | 8768747 | Yes |
| *Zscan26* | 13 | - | 21442180 | 21453730 | No |
| *Dsp* | 13 | + | 38151294 | 38198577 | Yes |
| *Rbm24* | 13 | + | 46418434 | 46431095 | No |
| *Rgs14* | 13 | + | 55369732 | 55384687 | Yes |
| *Ntrk2* | 13 | + | 58806569 | 59133970 | No |
| *Dapk1* | 13 | + | 60601947 | 60763191 | Yes |
| *Adcy2* | 13 | - | 68620043 | 68999541 | No |
| *Srd5a1* | 13 | - | 69573449 | 69611442 | Yes |
| *Pcsk1* | 13 | + | 75089826 | 75134861 | No |
| *Enc1* | 13 | + | 97241105 | 97253034 | Yes |
| *Arhgef28* | 13 | - | 97899469 | 98206439 | Yes |
| *Pik3r1* | 13 | - | 101680563 | 101768217 | No |
| *Ankrd55* | 13 | + | 112288451 | 112384002 | No |
| *Anxa7* | 14 | - | 20455260 | 20480133 | No |
| *Cacna1d* | 14 | - | 30039939 | 30491455 | Yes |
| *Itih3* | 14 | - | 30908572 | 30923760 | No |
| *Fbxo34* | 14 | + | 47450421 | 47531962 | No |
| *Fdft1* | 14 | - | 63145150 | 63179578 | No |
| *Ptk2b* | 14 | - | 66153257 | 66281052 | Yes |
| *Bmp1* | 14 | - | 70474558 | 70520234 | No |
| *Lmo7* | 14 | + | 101729957 | 101934710 | Yes |
| *Dock9* | 14 | - | 121542046 | 121797837 | Yes |
| *Ubac2* | 14 | + | 121878620 | 122021034 | No |
| *Mtmr12* | 15 | + | 12205028 | 12274496 | Yes |
| *Cmbl* | 15 | + | 31565389 | 31590119 | No |
| *Sema5a* | 15 | + | 32244810 | 32696341 | Yes |
| *Kcns2* | 15 | + | 34837355 | 34843407 | Yes |
| *Mtss1* | 15 | - | 58941234 | 59082005 | Yes |
| *Nrbp2* | 15 | - | 76085518 | 76091626 | Yes |
| *Parvb* | 15 | + | 84232043 | 84315688 | No |
| *Rabl2* | 15 | - | 89582533 | 89591923 | No |
| *Nell2* | 15 | - | 95219451 | 95528706 | Yes |
| *Tmem106c* | 15 | + | 97964229 | 97970286 | No |
| *Rnd1* | 15 | - | 98663421 | 98677461 | Yes |
| *Krt77* | 15 | - | 101858732 | 101869705 | No |
| *Ppp1r1a* | 15 | - | 103530279 | 103538003 | No |
| *Trap1* | 16 | - | 4039971 | 4077827 | Yes |
| *Srl* | 16 | - | 4480216 | 4541816 | No |
| *Tmem114* | 16 | - | 8409276 | 8425136 | No |
| *Mkl2* | 16 | + | 13256481 | 13417529 | Yes |
| *Acap2* | 16 | - | 31092413 | 31201238 | Yes |
| *Kalrn* | 16 | - | 33974258 | 34573532 | Yes |
| *Zbtb20* | 16 | + | 42875881 | 43642602 | Yes |
| *Zdhhc23* | 16 | - | 43969078 | 43979093 | No |
| *Robo1* | 16 | + | 72663149 | 73046095 | No |
| *Robo2* | 16 | - | 73892306 | 74411825 | No |
| *App* | 16 | - | 84949685 | 85173766 | Yes |
| *Tiam1* | 16 | - | 89787111 | 89980080 | Yes |
| *Acat2* | 17 | - | 12942890 | 12960747 | No |
| *Smoc2* | 17 | + | 14279506 | 14404790 | Yes |
| *Cacna1h* | 17 | - | 25374285 | 25433783 | Yes |
| *Narfl* | 17 | + | 25773776 | 25783332 | No |
| *Btbd9* | 17 | - | 30215524 | 30576287 | Yes |
| *Pde9a* | 17 | + | 31386234 | 31476310 | Yes |
| *Cul7* | 17 | + | 46650337 | 46664364 | No |
| *Dlgap1* | 17 | + | 69969073 | 70821413 | Yes |
| *Prkce* | 17 | + | 86167785 | 86657919 | Yes |
| *Mkx* | 18 | - | 6910459 | 7004780 | Yes |
| *Abhd3* | 18 | - | 10644411 | 10706771 | Yes |
| *Osbpl1a* | 18 | - | 12755314 | 12941841 | Yes |
| *Garem1* | 18 | - | 21127201 | 21300138 | Yes |
| *Nrg2* | 18 | - | 36017707 | 36197380 | No |
| *Arhgap26* | 18 | + | 38601534 | 39376284 | No |
| *Prr16* | 18 | + | 51117898 | 51304641 | No |
| *Zfp608* | 18 | - | 54888045 | 54990180 | No |
| *Nedd4l* | 18 | + | 64887705 | 65217831 | Yes |
| *Cidea* | 18 | + | 67343564 | 67367794 | No |
| *Tcf4* | 18 | + | 69343356 | 69689079 | Yes |
| *B4gat1* | 19 | + | 5038826 | 5041134 | No |
| *Rin1* | 19 | + | 5050481 | 5057071 | Yes |
| *Rcor2* | 19 | + | 7267325 | 7275225 | No |
| *Mta2* | 19 | + | 8941875 | 8952303 | No |
| *Lrrc10b* | 19 | - | 10455371 | 10457447 | Yes |
| *Tmem138* | 19 | - | 10570478 | 10577362 | Yes |
| *Aldh1a1* | 19 | + | 20492715 | 20643462 | No |
| *Klf9* | 19 | + | 23141226 | 23166911 | No |
| *Rbp4* | 19 | - | 38116620 | 38125321 | No |
| *Sfxn3* | 19 | + | 45047576 | 45056383 | Yes |
| *Actr1a* | 19 | - | 46376814 | 46395735 | No |
| *Tmem255a* | X | - | 38196573 | 38252439 | No |
| *Gpc4* | X | - | 52053021 | 52165252 | No |
| *Pnck* | X | - | 73655994 | 73660117 | No |
| *L1cam* | X | - | 73853778 | 73896105 | No |
| *Atp6ap1* | X | + | 74297097 | 74304721 | Yes |
| *Plxna3* | X | + | 74329066 | 74344689 | No |
| *Arx* | X | + | 93286445 | 93298357 | No |
| *Slc7a3* | X | - | 101079210 | 101086020 | Yes |
| *Rtl5* | X | - | 102066544 | 102071304 | No |
| *Magt1* | X | - | 105968084 | 106011906 | No |
| *Armcx6* | X | - | 134748461 | 134751417 | No |
| *Gprasp2* | X | + | 135839034 | 135844730 | No |
| *Vegfd* | X | + | 164373378 | 164402650 | No |
| mmu-miR-3095-5p | 4 | - | 58441064 | 58441085 | No |
| mmu-miR-150-5p | 7 | + | 45121762 | 45121783 | No |
| mmu-miR-24-1-5p | 13 | + | 63301213 | 63301235 | No |
| mmu-miR-1943-5p | 15 | - | 79375271 | 79375293 | No |

Notes: 1) Chr: Chromosome; 2) Weight: the corresponding topological overlap of genes with mmu-miR-190a-3p are great than 0.05.
