# Supplementary material for: MiR-190a potentially ameliorates postoperative cognitive dysfunction by regulating Tiam1
Source: BMC Genomics. 2019 Aug 22;20:670. doi: 10.1186/s12864-019-6035-0 (PMC6704709; doi:10.1186/s12864-019-6035-0)
Supplement: Supplementary file 2 — Figure S1. The detailed time line of the euthanization of study animals. According to the protocol, all mice were euthanized before obtaining hippocampal tissue. All mice were deeply anesthetized based on several signs (see Methods part). Then the mice were decapitated 24 h after operation, brains were instantly dissected on ice, and the hippocampal tissues were obtained and stored in liquid nitrogen. Figure S2. Volcano plot of miRNA expression in replication cohort. This dataset (GSE95070) was downloaded from the GEO database. Figure S3. The results of qRT-PCR for technical replication. Expression level of the most significantly mmu-miR-190a-3p was validated by using real-time PCR assay. Reverse transcription reaction was performed with M-MLV Reverse Transcriptase kit (Takara Code: D2639A) based on the manufacturers’ protocol. Figure S4. WGCNA module-based analysis for genes and miRNAs expression data. The genes and miRNAs expression data of GSE73507 were acquired from GEO database. After the filtering process, 13,241 mRNAs and 546 miRNAs from hippocampus tissue of wild type mice (n = 24) were included for WGCNA analysis. Figure S5. KEGG pathway analyses for mmu-miR-190a-3p highly related genes in red module. We refined 169 genes from red module with a highly connected with mmu-miR-190a-3p (Fig. 2b) and used ClueGO (v. 2.3.4) to decipher the pathways and determine their biological functions. (PPTX 118 kb) [file 12864_2019_6035_MOESM2_ESM.pptx]

## Slide 1
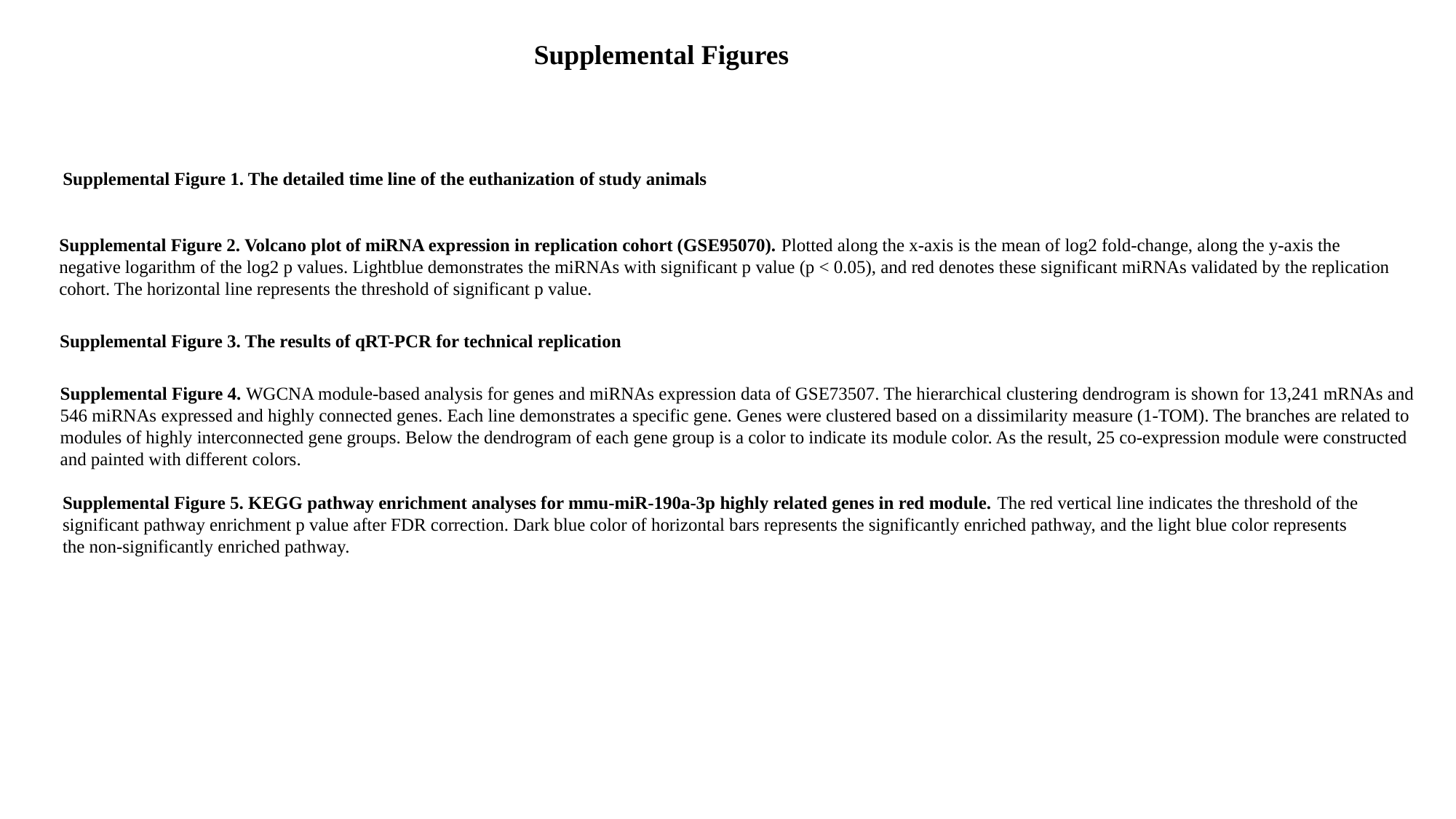

Supplemental Figures
Supplemental Figure 1. The detailed time line of the euthanization of study animals
Supplemental Figure 2. Volcano plot of miRNA expression in replication cohort (GSE95070). Plotted along the x-axis is the mean of log2 fold-change, along the y-axis the negative logarithm of the log2 p values. Lightblue demonstrates the miRNAs with significant p value (p < 0.05), and red denotes these significant miRNAs validated by the replication cohort. The horizontal line represents the threshold of significant p value.
Supplemental Figure 3. The results of qRT-PCR for technical replication
Supplemental Figure 4. WGCNA module-based analysis for genes and miRNAs expression data of GSE73507. The hierarchical clustering dendrogram is shown for 13,241 mRNAs and 546 miRNAs expressed and highly connected genes. Each line demonstrates a specific gene. Genes were clustered based on a dissimilarity measure (1-TOM). The branches are related to modules of highly interconnected gene groups. Below the dendrogram of each gene group is a color to indicate its module color. As the result, 25 co-expression module were constructed and painted with different colors.
Supplemental Figure 5. KEGG pathway enrichment analyses for mmu-miR-190a-3p highly related genes in red module. The red vertical line indicates the threshold of the significant pathway enrichment p value after FDR correction. Dark blue color of horizontal bars represents the significantly enriched pathway, and the light blue color represents the non-significantly enriched pathway.

## Slide 2
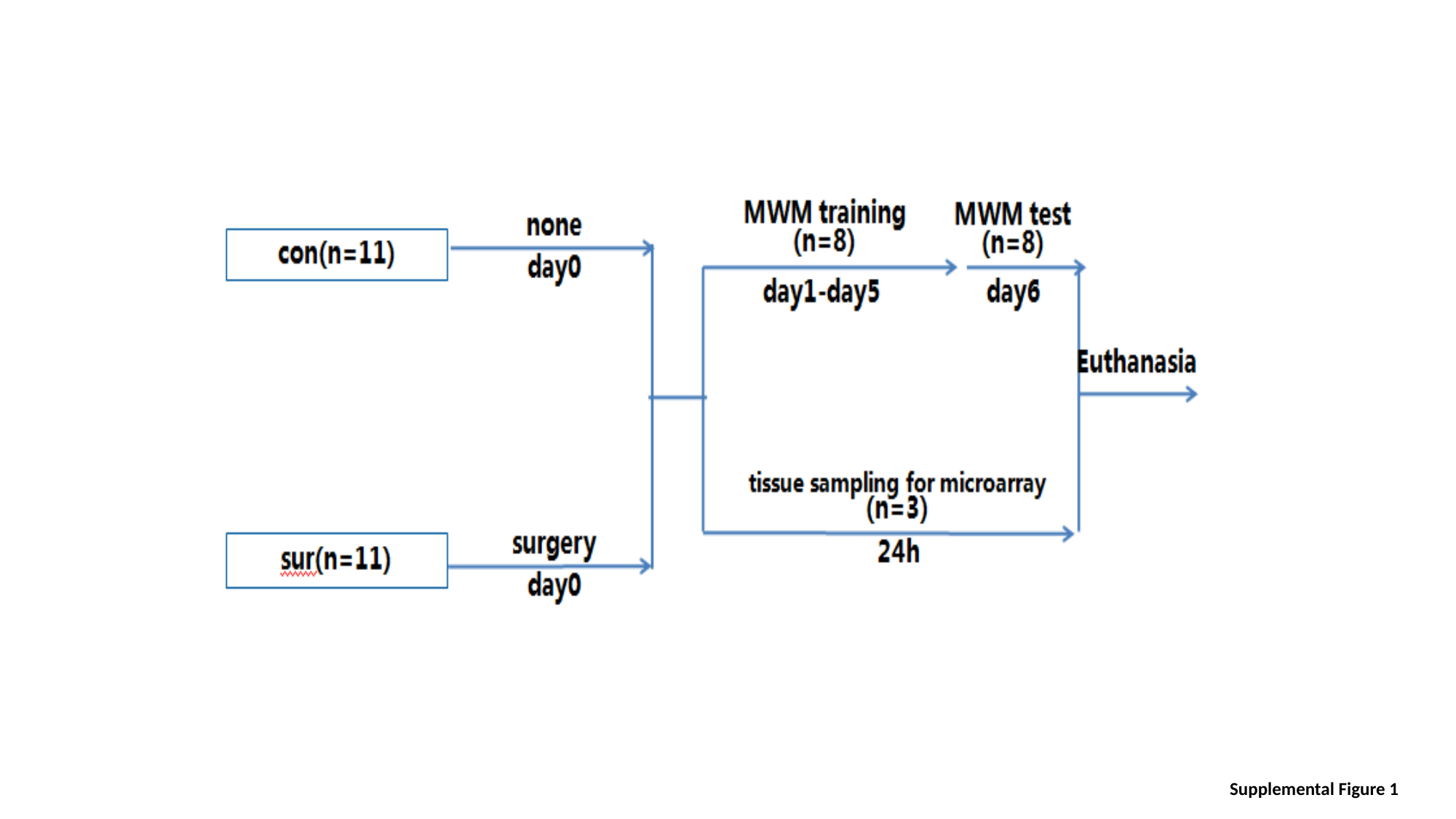

Supplemental Figure 1

## Slide 3
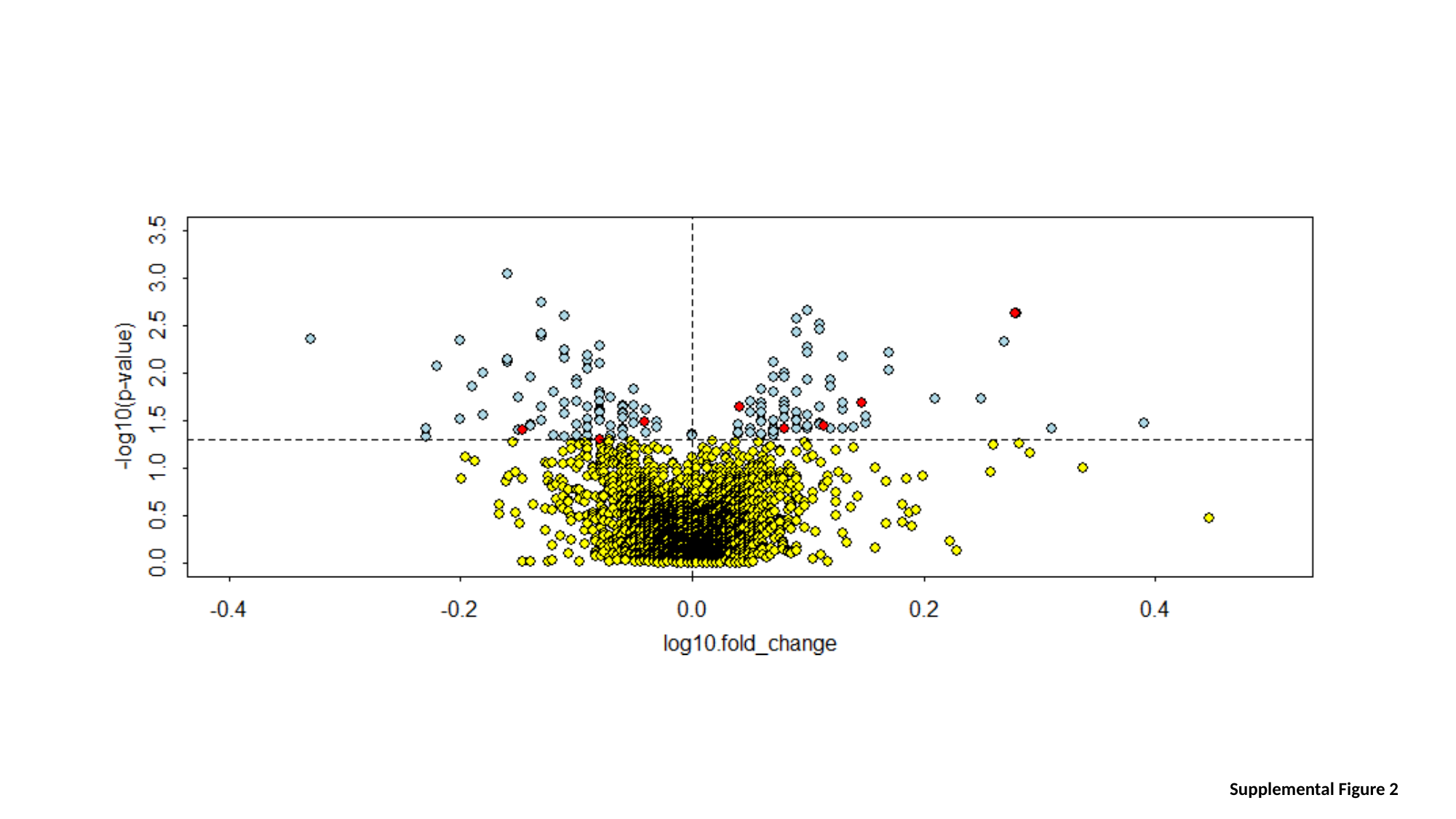

Supplemental Figure 2

## Slide 4
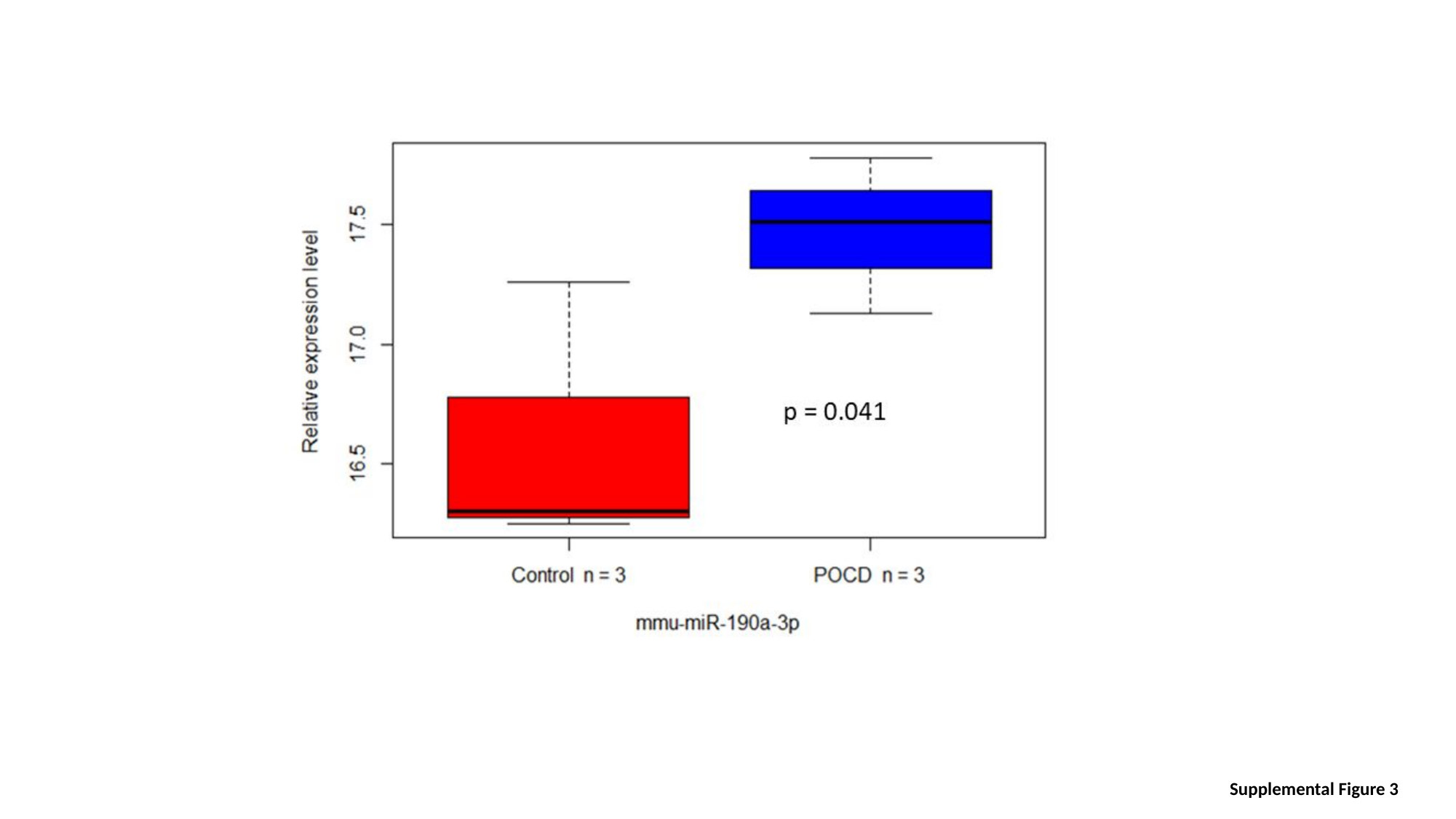

Supplemental Figure 3

## Slide 5
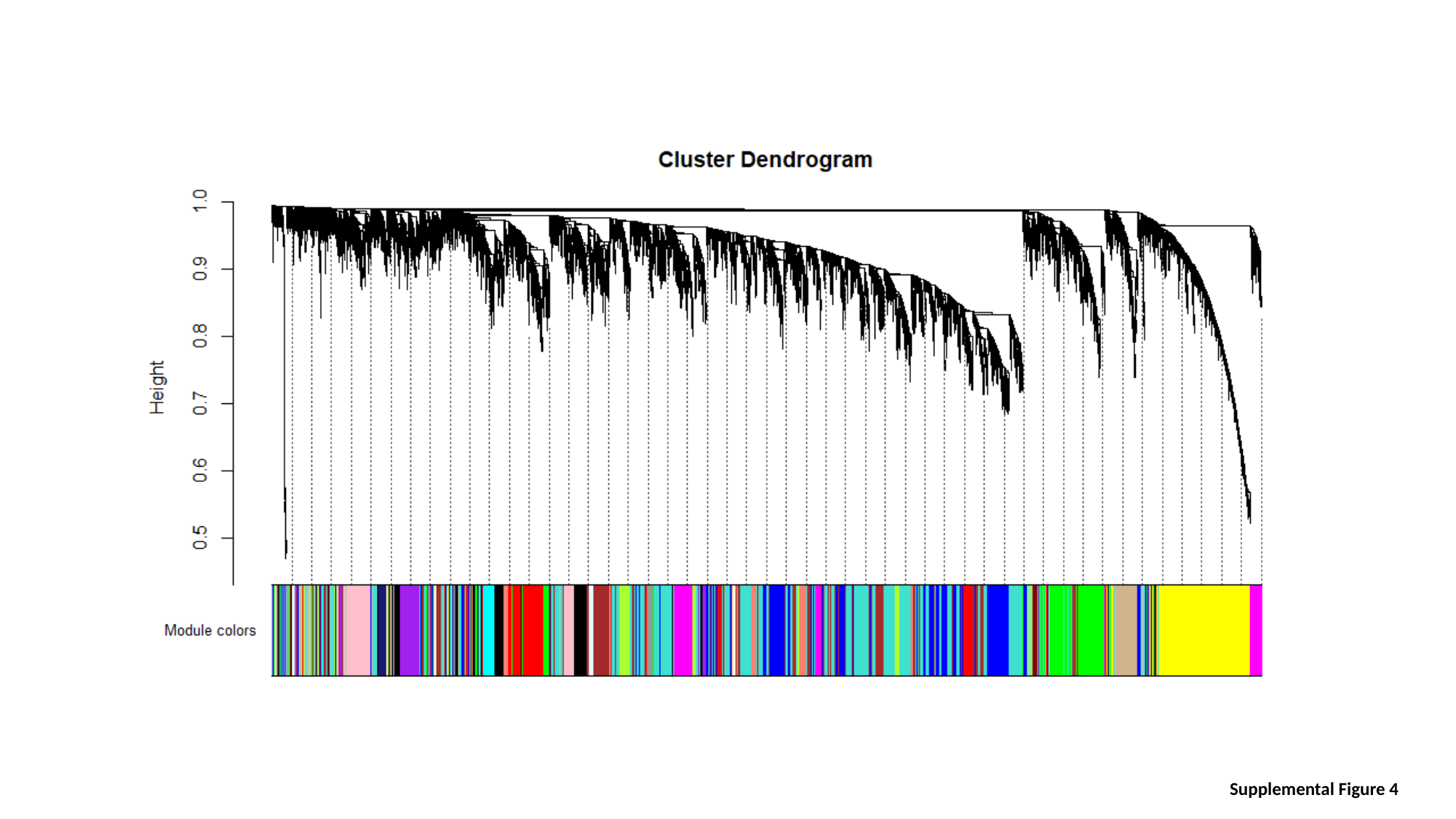

Supplemental Figure 4

## Slide 6
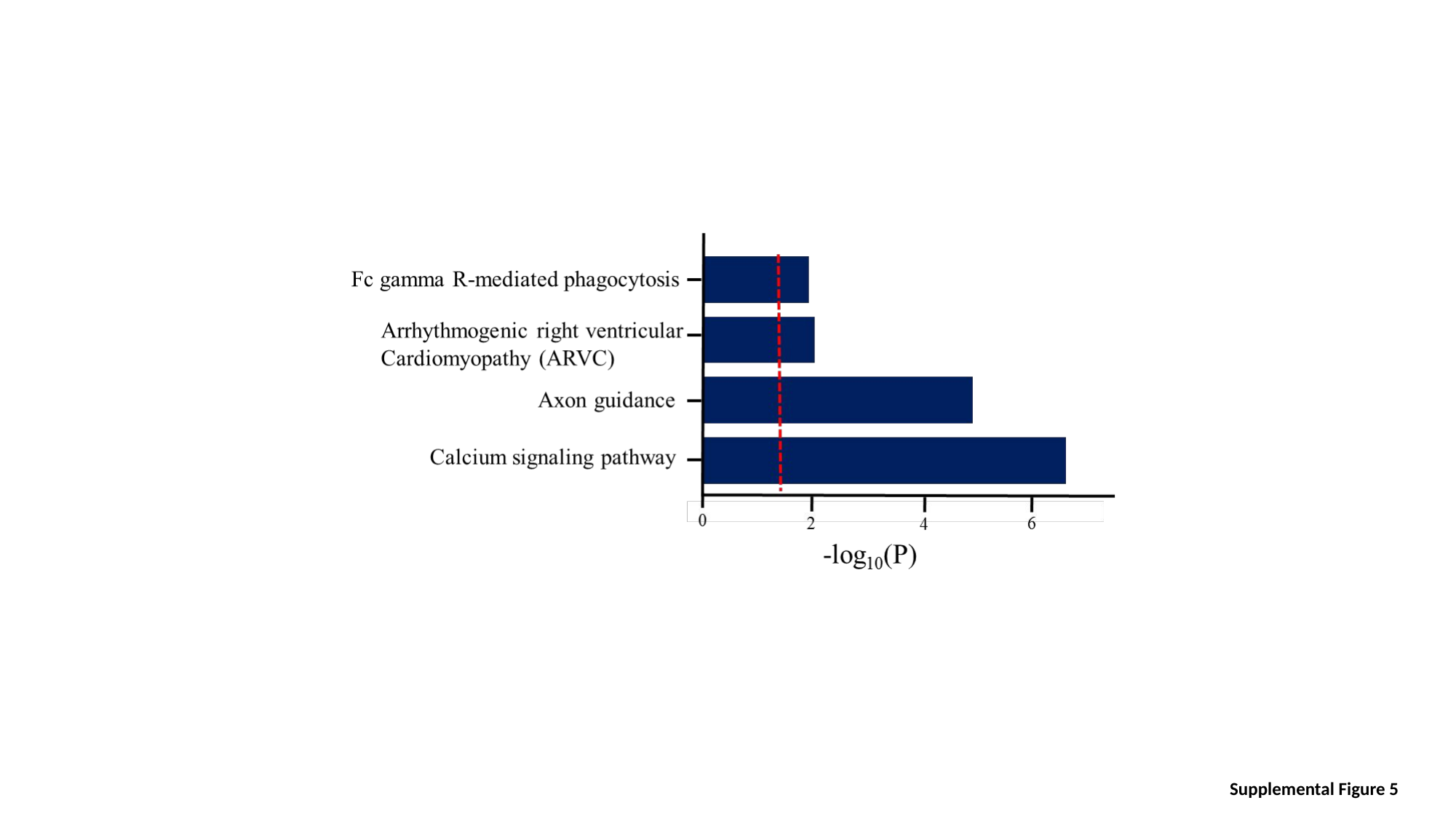

Supplemental Figure 5
